# Psychosocial Impact of Maxilla-For-All^®^ Treatment Using Standard and Long Implants (Pterygoid, Trans-Sinus and Zygomatic) on Patients with Severe Maxillary Atrophies: A 1-Year Prospective Study with PIDAQ-23 and OHIP-14

**DOI:** 10.3390/jcm14103544

**Published:** 2025-05-19

**Authors:** Tommaso Grandi, Paolo Toti, Cesare Paoleschi, Matteo Giorgi, Ugo Covani, Giovanni Battista Menchini-Fabris

**Affiliations:** 1 Independent Researcher, 41126 Modena, Italy; t.grandi@grandiclinic.com; 2 Independent Researcher, 55041 Camaiore, Italy; capello.totipaolo@gmail.com; 3 Independent Researcher, 50132 Florence, Italy; cesarepaoleschi@gmail.com; 4 Independent Researcher, 25123 Brescia, Italy; giorgidrmatteo@pec.it; 5 School of Dentistry, Saint Camillus International University of Health and Medical Sciences, Via di Sant’Alessandro, 8, 00131 Rome, Italy; covani@covani.it; 6 Department of Psychology and Health Sciences, University Pegaso, Business District, Isola F2, 80143 Napoli, Italy

**Keywords:** implant-supported full-arch prosthesis, severe maxillary atrophies, OHIP, PIDAQ, patient satisfaction, aesthetics, quality of life

## Abstract

**Background/Objectives**: The satisfaction of patients following maxillary full-arch rehabilitation is crucial in assessing treatment effectiveness. This one-year study evaluated patients’ satisfaction, quality of life, and aesthetic perception after receiving the Maxilla-for-All^®^/All-On-X treatments, which combine standard, pterygoid, trans-sinus, and zygomatic implants to support a fixed prosthesis and offer a graftless solution that reduces morbidity and treatment time. **Methods**: A prospective cohort study using convenience sampling of subjects treated for severe maxillary atrophies was conducted on patients receiving immediate implant-supported full-arch fixed prostheses. The Oral Health Impact Profile (OHIP-14) and Psychosocial Impact of Dental Aesthetics Questionnaire (PIDAQ-23) were administered preoperatively and one year post-treatment. Patients were grouped based on the presence or absence of complications (surgical, technical, and mechanical) and Wilcoxon tests were used for comparison (significance level = 0.05). **Results**: A total of 56 patients (29 female, 27 male) participated, with no implant or prosthesis failures. Eleven patients reported unilateral sinus membrane perforation, and seven had technical or mechanical complications. Preoperatively, 69% of patients rated their oral condition as unfavorable according to the OHIP-14; this dropped significantly to 21.8% post-treatment (*p*-value < 0.0001). After one year, the average PIDAQ-23 score improved significantly from 44.7 ± 16.6 to 6.8 ± 5.3 (*p*-value < 0.0001). No significant differences were observed between patients with or without complications (*p*-values ranging from 0.5270 to 0.8920). **Conclusions**: Full-arch rehabilitation using Maxilla-for-All^®^/All-On-X treatments significantly improved both aesthetic perception and chewing function in patients with severe maxillary atrophies. They reported a substantial reduction in oral health-related discomfort, as shown by a significant decrease in OHIP-14 scores one year post-treatment. Clinical or technical complications did not significantly impact patients’ quality-of-life outcomes or satisfaction, supporting the reliability of this treatment protocol.

## 1. Introduction

With the advent of implant-supported restorations, the rehabilitation of chewing function and aesthetics in patients who are long-standing partially or totally edentulous has been transformed and now goes beyond the traditional removable dentures, which are increasingly used as a staged approach to replace fixed dental prostheses [1]. It is difficult for clinicians to restore posterior edentulous maxillae with implants because of the low quality and quantity of residual jawbone and reduced implant primary stability; moreover, when facing wide bone atrophies, they encounter several difficulties in placing enough implants to adequately support the prostheses [2,3].

In patients with severe maxillary atrophies, the rehabilitation of edentulism with conventional implants supporting fixed restorations usually involves sinus floor augmentation, bone grafting, and subsequent soft tissue management [4]. These treatments often result in increased morbidity rates due to surgical issues, such as graft failure or infection, and lead to longer recovery times and expenses, which encourage clinicians to explore other rehabilitation strategies [5]. The need to insert implants in areas with reduced bone volume and/or density, especially in posterior maxillary regions, drives clinicians to seek alternative ways to utilize the existing bone by using short implants [6,7]. To overcome the major limitations of severe maxillary bone atrophy, the Maxilla-for-All^®^ approach, which defines an implant-supported fixed-hybrid prosthesis (or all-on-X rehabilitation strategy), offers an alternative to major reconstructive procedures for atrophic maxillary ridges [8]. The Maxilla-for-All^®^ protocol aims to treat patients with severe maxillary atrophy with an immediate graftless solution that combines standard axial implants in the premaxilla with pterygoid, trans-sinus, and/or zygomatic implants in the posterior regions where the bone available is insufficient to place standard implants alone.

Using longer implants, it becomes possible to reach the residual maxillary bone cortical pillars (pterygoid, zygomatic, and canine pillars), leading to high primary implant stability and allowing an immediate loading rehabilitation.

Implant-supported full-arch prostheses, which are currently considered very predictable rehabilitation strategies, involve immediate implant loading [9,10].

This study represented an initial longitudinal investigation—albeit limited to the short term—that offered a comprehensive and detailed analysis of multiple patient-reported satisfaction indices following full-arch prosthetic rehabilitation. Despite the increasing clinical use of the mixed approaches (Maxilla-for-All^®^/All-On-X), previous research had quantitatively assessed patient-reported outcomes such as satisfaction, aesthetics, and quality of life following fixed rehabilitation supported by standard dental implants. Studies have shown that patients who receive immediately loaded implant-supported prostheses express a very high degree of satisfaction [11]. In fact, the immediate loading approach in full-arch rehabilitations shortens treatment times and offers patients several psychological benefits; all these factors make implant treatments more appealing [12]. To the best of our present knowledge, this was among the first longitudinal studies to evaluate these outcomes while also considering the potential impact of surgical and technical complications on full-arch rehabilitation.

The OHIP-14 and PIDAQ-23 are validated and widely accepted instruments for assessing oral health-related quality of life and the psychosocial impact of dental aesthetics, respectively. Their application has been demonstrated in studies evaluating patient satisfaction after implant-supported fixed prostheses using standard root-form implants [11,13,14]. These tools allowed for standardized comparisons and were sensitive to changes in functional and aesthetic outcomes. Their use in the present study enabled a consistent and comprehensive evaluation of patient-reported outcomes following full-arch rehabilitation strategies supported by different types of implants.

Null Hypothesis (H_0_): There is no statistically significant difference in quality-of-life outcomes, as measured by OHIP-14 and PIDAQ-23 scores, between patients who experience early complications and those who do not following Maxilla-for-All^®^/All-On-X prosthetic rehabilitation. Using a combination of standard and long implants to support full-arch immediate prostheses, the present study aims to determine patients’ satisfaction and aesthetic perception with the Maxilla-for-All^®^ treatment concept. A preliminary one-year survey was conducted. Comparing patient satisfaction and quality of life between those who experience surgical, mechanical, or technical complications and those who do not constituted the secondary goal.

## 2. Materials and Methods

### 2.1. Subjects

This research was designed as a single-arm prospective cohort study with a one-year follow-up period. The study was conducted across three private dental centers located in Modena, Florence, and Brescia, involving multiple experienced clinicians who followed a standardized surgical and prosthetic protocol. A total of 56 patients were included through convenience sampling; no formal sample size calculation or power analysis was performed, as the primary objective was exploratory and focused on capturing preliminary patient-reported outcomes.

The study, conducted from 2022 to 2023, aimed to assess satisfaction and quality of life of patients requiring full-arch rehabilitation supported by standard, trans-sinus, pterygoid, and zygomatic implants. It was carried out across three private dental centers located in Modena, Florence, and Brescia involving multiple experienced clinicians who followed a standardized surgical and prosthetic protocol. Patients receiving immediate implant-supported full-arch fixed prostheses were included through convenience sampling; no formal sample size calculation or power analysis was performed, as the primary objective was exploratory and focused on capturing preliminary patient-reported outcomes.

Treated subjects were ranked according to the presence or absence of surgical, mechanical, or technical complications.

Before implant placement, patients received an invitation to take part in a recall program, and clinicians administered two questionnaires to all patients to assess their satisfaction levels. The OHIP-14 and PIDAQ-23 questionnaires—validated instruments for assessing oral health-related quality of life and dental aesthetic satisfaction—were administered in their officially translated Italian versions. Surveys were self-administered by patients in a standardized clinical setting to minimize interviewer bias; however, blinding was not complete due to the nature of the intervention and follow-up assessments. Patients were called back for re-examination at the San Rossore Dental Unit (affiliated unit of the Tuscan Stomatological Institute). Clinically relevant and available parameters were retrospectively gathered, entered into a database, and prepared for statistical software analysis.

Explicit consent was obtained for the use of patients’ clinical information, and all procedures performed in studies involving human participants were in accordance with the ethical standards of the institutional and/or national research committee and with the 1964 Helsinki Declaration and its later amendments. The present analysis was approved by the Regional Ethical Review Board of Saint Camillus International University of Health Sciences (under reference number ID: E00930-2023).

#### 2.1.1. Inclusion Criteria

Each patient needed to meet the following criteria to be included:

- Available radiographs before implant placement and 12 months after implant loading;
- Treatments with immediate functional loading of the implants and full-arch rehabilitation supported by standard, trans-sinus, pterygoid, and zygomatic implants;
- Age ≥ 18 years at surgery;
- Completed questionnaires.

#### 2.1.2. Exclusion Criteria

Patients were removed from the analysis after being included if they met any of the following criteria:

- Undergoing any augmentation surgery;
- Medical records showing a history of systemic diseases that would have contraindicated oral surgery;
- Using bisphosphonate medications;
- Receiving bone resection or radiation therapy;
- Experiencing emotional instability;
- Abusing drugs or alcohol;
- Smoking heavily (more than 20 cigarettes a day).

### 2.2. Instrumentation/Surgery

Preoperative clinical history and radiographic examinations (Computed Tomography [TC] and Orthopantomography [OPG]) were carried out for every patient as required by current best practice. Before implant surgery, 1 g of amoxicillin plus clavulanic acid (or 500 mg of clarithromycin in the case of a penicillin allergy) was used for antimicrobial prophylaxis. This began the evening before the surgical procedure and was administered twice a day for the next 7 days after surgery. Every patient received local anesthesia and intravenous sedation during their surgical procedures. The anesthetic was injected into the buccal sites between the central incisor and the third molar; then, when required for the procedure, bilateral blocks of the infraorbital and palatal posterior nerves were performed. The height and volume of the buccal bone were preserved for each tooth to be extracted by minimizing trauma during removal. Alveolar process remodeling was not required in almost all cases.

**Figure 1:**
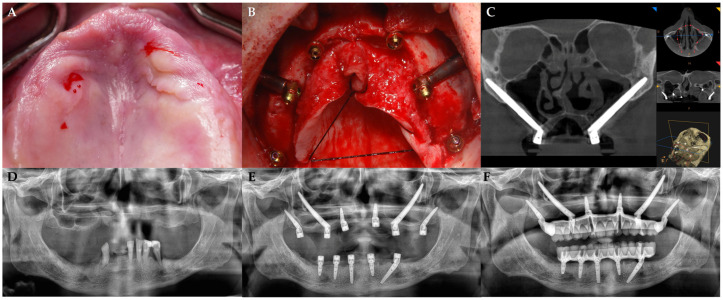
(**A**) Preoperative clinical view. (**B**) Intraoperative view after flap elevation and placement of implants. (**C**) Postoperative CT scans with 3D reconstruction showing the trajectory and position of zygomatic implants with A—Anterior; P—Posterior; R—Right; L—Left; H—Head; F—Foot. (**D**) Preoperative orthopantomogram. (**E**,**F**) Postoperative orthopantomograms showing the implants in place (**E**) and the final prosthetic rehabilitation (**F**). The abutments were attached following flap closure with non-resorbable sutures (4/0). The following post-surgical instructions were given: (1) ibuprofen 400 mg (or paracetamol 1 g for patients allergic to NSAIDs) to be taken 2–4 times a day, during meals if needed; (2) 0.2% chlorhexidine mouthwash for 1 min twice a day for 2 weeks. A soft diet was recommended for 3 weeks.

Following the manufacturer’s instructions, patients were treated with anterior axial tapered implants with internal connection (JDEvolution S, JDEvolution Plus, JDIcon, and JDIcon Plus, JDentalCare, Modena, Italy) or with trans-sinus implants (JDNasal, JDentalCare, Modena, Italy). Zygomatic implants could be used to treat the patients’ posterior areas. Briefly, after a full-thickness flap elevation and exposure of the alveolar bone, lateral maxillary wall, and underside area of the zygoma prominence, an adequate implant bed carved along the external cortex of the lateral sinus wall was performed. Drilling through the cancellous layer was performed up to the depth indicated during the planning stage. Using a pilot/twist drill set, a deep groove was drilled (from 2.8 mm to 3.6 mm). Finally, the implant body was manually placed within the groove, while the apex was screwed to the zygomatic bone, achieving an extrasinus approach (JDZygoma, JDentalCare, Modena, Italy). Thus, the implant’s head emerged at the top of the alveolar crest [15]. The following procedure was used when a pterygoid implant placement was deemed necessary: a crestal incision was made and a full-thickness flap was elevated; implants were then positioned in the maxilla’s pterygoid regions. To obtain an insertion torque of at least 45 Ncm before the implant’s final seating (measured by using a calibrated torque wrench), an under-preparation protocol was adopted (Figure 1). Implants were positioned in the pterygomaxillary region at about a 25–40 degree angle to the maxillary plane (JDPterygo, JDentalCare, Modena, Italy), according to Graves’ technique [16].

All patients were treated with a temporary fixed implant-supported prosthesis. The immediate interim hybrid full-arch screw-retained prostheses were supported by universal conical abutments on different dental implants of the same brand, including standard dental implants plus zygomatic, pterygoid, and trans-sinus implants, which were positioned vertically or tilted according to the surgeon’s judgment and the quantity of residual and available bone tissue. The final prosthesis was delivered six months after surgery.

All patients were subjected to a strict oral and implant hygiene maintenance protocol. Each participant was enrolled in a recall program and monitored regularly by a professional dental hygienist throughout the follow-up period. This included professional prophylaxis sessions, reinforcement of individual oral hygiene instructions, and clinical checks to ensure tissue health.

### 2.3. Measurement

The descriptive variables of the sample were age, gender, and implant type.

#### 2.3.1. Predictors

Information related to the presence (YES) or absence (NO) of complications, such as biological and mechanical ones, was gathered for each patient.

#### 2.3.2. Primary

- OHIP-14: A short version of the OHIP 49-item questionnaire suggested by Slade and co-workers is called the OHIP-14 [17]. Its seven domains are the following: functional limitation, physical pain, psychological discomfort, physical disability, psychological disability, social disability, and handicap. The Oral Health Impact Profile-14 comprises a total of 14 items rated on a scale of 0–4 (0 = “never”, 1 = “rarely”, 2 = “occasionally”, 3 = “often”, 4 = “very often”).
- The Psychosocial Impact of Dental Aesthetics Questionnaire (PIDAQ), a psychometric tool developed by Klages, is used to evaluate aspects of quality of life specific to orthodontics [18]. The questionnaire assesses the influence of aesthetic perception of teeth in daily life and consists of 23 items grouped into four factors: dental self-confidence (DSC), social impact (SI), psychological impact (PI), and aesthetic concern (AC). The responses to aesthetic-negative statements were rated on a scale of 0–4 (0 = “not at all”, 1 = “a little”, 2 = “somewhat”, 3 = “strongly”, or 4 = “very strongly”). Since the subgroup “dental self-confidence” consisted of aesthetic-positive statements, the scale was reverse-coded for the first component. Thus, a total score of 0 would represent absolute satisfaction with aesthetics, and a maximum total score of 92 would represent absolute dissatisfaction.

#### 2.3.3. Secondary

- Biological: Clinical examination of sinusitis symptoms and signs using the Lanza–Kennedy score assessed how surgery, implant placement, and potential sinus membrane perforation affected the ipsilateral upper respiratory tract (ear, nose, and throat) [19]. A criterion for soft tissue infection or inflammation was the appearance of clinical symptoms, infection, and dehiscence following implant implantation.
- Mechanical or Technical: Any fractures (generally including chipping of the veneering materials, components, frameworks, and structures) could be the cause of mechanical issues with an implant abutment or prosthesis. Prosthesis detachment, decementation, debonding, or loss of a retention screw were defined as technical issues [20].

### 2.4. Statistical Procedures

Data were loaded by software for data processing and statistical analysis (MatLab 7, The MathWorks, Inc., Natick, MA, USA). The statistical unit of the analysis was the patient. A single investigator with expertise in dental biostatistics retrospectively analyzed all the data (TP). A descriptive analysis with mean, standard deviation, median, interquartile range, minimum, and maximum for continuous data was conducted. Homoscedasticity was tested but not verified by the Brown–Forsythe test. The Gaussian distribution was not confirmed by the Shapiro–Wilk analysis. A nonparametric two-way ANalysis Of VAriance (Friedman ANOVA test) was used to study the effect of subgroups on continuous measurement variables (OHIP-14 or PIDAQ-23). Independent groups were pair-compared by the Wilcoxon rank-sum test. Measurements repeated in time (preoperative versus postoperative) were pair-compared using the Wilcoxon signed-rank test. The level of significance was set at 0.05 for all the analyses.

## 3. Results

A total of 56 patients (29 female and 27 male) with a mean age of 65 ± 7.4 years were included in the present study. At baseline, all included patients suffered from long-standing maxillary edentulism in the posterior areas, leading to alveolar ridge resorption, which in many cases necessitated the use of non-conventional implant placement. Patients received at least four implants and immediate full-arch rehabilitations. In total, 297 implants were placed across the study population, including 73 zygomatic, 115 standard, 92 pterygoid, and 17 trans-sinus implants, with each patient receiving between 4 and 7 implants, resulting in an average of approximately 5.3 implants per patient. Neither implant nor prosthesis failure were registered during the selected time interval. None of the variable distributions were Gaussian, so nonparametric analyses were required.

Patients were ranked into groups based on whether they had experienced either biological and mechanical issues or not. During surgical intervention, 11 patients were reported to have unilateral sinus membrane perforation, whereas technical or mechanical issues were mentioned in 7 patients. There were no differences observed when post hoc testing for multiple comparisons was applied to the three groups of patients: those with negative surgical reports, those with technical or mechanical issues, and those with neither of them.

Table 1 provides a summary of all patient questionnaires’ responses by items and factors.

Results of the ANOVA tests indicated that gender (male versus female) had no statistically significant influence on both the postoperative OHIP-14 and PIDAQ-23 questionnaires.

### 3.1. OHIP

The descriptive statistics of the answers to the Oral Health Impact Profile questionnaire are shown in Table 1. Overall, 69% percent of patients evaluated their preoperative oral condition as unfavorable for health; one year after surgery and oral immediate rehabilitation, the percentage significantly dropped to 21.8%. The postoperative OHIP items (with a mean score of 12.0 ± 6.4) appeared to be different from the preoperative OHIP items (with a mean score of 27.0 ± 7.6) in a very statistically significant way (*p*-value < 0.0001), as shown in Table 2. Significance in bold.

This supported the conclusion that therapy with immediate full-arch rehabilitation could be medical care that is very beneficial for patients suffering from severe posterior bone atrophy, at least. Again, when comparing the preoperative and postoperative status of the patients, items about functional discomfort (that is, functional limitation, physical pain, physical disability, and handicap) revealed a net decrease in unfavorable conditions, from 68.1% to 31.7% of patients, respectively.

**Figure 2:**
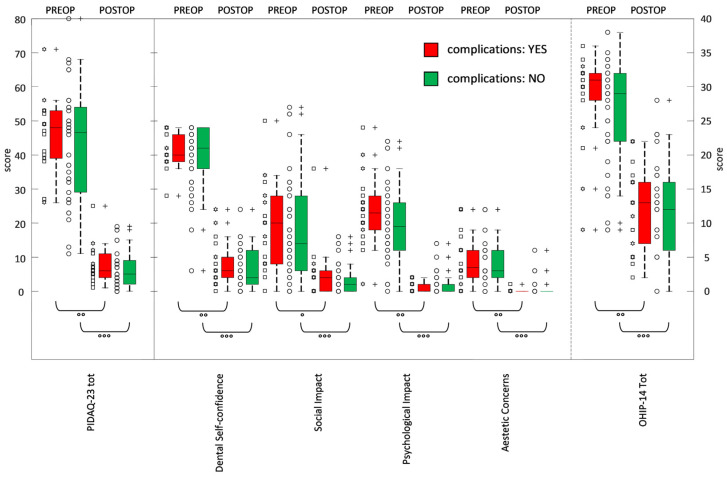
Box and whisker plot of the PIDA-23 (with 4 components) and OHIP-14 at baseline (preoperative or PREOP) and at 12 months (postoperative or POSTOP). Pair-wise statistical comparisons: Wilcoxon signed-rank test assessed changes in time: significant (° with 0.001 ≤ *p*-values < 0.01), very significant (°° with 0.0001 ≤ *p*-values < 0.001), extremely significant (°°° with *p*-values < 0.0001). ◯: no complications; +: outliers lying in both extremes of data; ☐: medical complications; 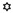: mechanical complications.

When the answers of patients with or without complications of any kind were compared, no significant differences were found between the two groups (Table 3). The postoperative total scores of groups with and without complications were 12.2 ± 6.1 and 12.0 ± 6.6, respectively—values that were very similar. Detailed scatter plots illustrating the distribution of OHIP scores among the three groups (surgical complications, technical/mechanical issues, and no complications) are presented in Figure 2.

### 3.2. PIDAQ

The frequencies of the answer categories for all 23 questions are summarized in Table 1. The average Psychosocial Impact Of Dental Aesthetics Questionnaire score of all 56 patients was 44.7 ± 16.6 at the preoperative time and 6.8 ± 5.3 one year after rehabilitation. These values were significantly different (*p*-value < 0.0001), as shown in Table 2. Analyzing the factors a little bit more deeply, the mean scores of reverse dental self-confidence were 20.2 ± 4.3 before surgery and 3.5 ± 3.0 one year later, indicating a significant difference. Statements across each factor clearly reflected a post-treatment improvement in dental self-perception. Both social impact and psychological impact, with preoperative scores of 9.4 ± 7.2 and 10.5 ± 5.7, respectively, declined significantly to 2.1 ± 3.0 and 1.0 ± 1.3 postoperatively. Finally, the mean score for aesthetic concern, which was typically the main issue that patients with long-term edentulism sought to address as quickly as possible, changed from a preoperative score value of 4.7 ± 3.6 to 0.3 ± 1.0, showing that, despite a statistically significant improvement for all the scores, patients still sought solutions to their aesthetic concerns, just like they would have for any other functional or social issues. When the answers of patients with or without complications of any kind were compared, no significant differences were found between the two groups (Table 3). The postoperative total scores of groups with and without complications were 7.5 ± 5.7 and 6.5 ± 5.1, respectively, and appeared to be very similar. Detailed scatter plots illustrating the distribution of PIDAQ scores among the same three groups are presented in Figure 2.

## 4. Discussion

Since all fixed prosthetic rehabilitation techniques supported by osseointegrated dental implants are currently highly safe and reliable, it has become necessary to use adjunctive success criteria that place a greater emphasis on the functional and aesthetic satisfaction of patients. Therefore, two different scales—PIDAQ-23 and OHIP-14—were employed in the present study to assess patient satisfaction before surgery and then twelve months after immediate prosthetic rehabilitation.

According to published research, rehabilitation strategies combining standard root-form implants placed in the anterior areas with pterygoid, trans-sinus, and/or zygomatic implants are now considered reliable and repeatable procedures with a very high level of success. The evident decrease in dissatisfaction could be associated with the positive reception of this high-impact surgical and prosthetic rehabilitation approach in severely atrophied maxillae, in particular taking into account the fact that neither implants nor prostheses had failed and that the survival of implant–prosthetic restorations did not act as a confounding factor. In fact, according to multiple medium- and long-term studies on patients with severe maxillary bone atrophy who received similar treatments as described here, the survival rate of non-conventional implants ranges from 97% to 100%, whereas the results of standard implants vary from 96% to 100% [21,22,23].

Each patient in the present study showed a notable improvement in their chewing function and appearance, most likely as a result of the Maxilla-For-All^®^/All-On-X implant-prosthetic rehabilitation approach, regardless of post-surgical, technical, and mechanical complications. Both Factors 1 and 4 of the PIDAQ-23 questionnaire, that is, dental self-confidence and aesthetic concern, showed significant improvement between preoperative and postoperative time. Furthermore, comparing the preoperative and postoperative results, it was also observed that all OHIP-14 factors about the restoration of the chewing function, including handicap, physical pain, functional limitation, and physical disability, appeared to be significantly improved.

By analyzing the differences between the two subgroups more thoroughly, that is, the presence or absence of any complication, only 18 patients experienced either mechanical troubles, primarily reparable prosthesis fractures, or intraoperative complications, such as primary Schneiderian sinus membrane perforation. No severe post-surgical complications were observed in patients treated with full-arch prostheses. For patients who had undergone maxillary sinus surgery, the clinical onset of signs and symptoms of sinusitis was thought to be the most frequent postoperative complication. This was also the case when extrasinus techniques were employed, with sinusitis reported in 1.5% and 14% of cases [21,24,25]. Despite being generally solvable, minor mechanical and technical issues seemed to be relatively uncommon events below 8% over an estimated 1-year complication rate, as reported by some authors for both veneer wearing/chipping and retention-screw fracturing/loosening [26,27]. The results describing the early clinical and mechanical/technical complications of the present paper appeared to be in line with those of the previously cited studies.

The two patient populations presented here, ranked in groups with and without early biological and technical/mechanical complications, demonstrated similar behavior. In fact, there were no significant differences between the frequency distribution of the OHIP-14 and PIDAQ-23 scores. That is, both groups were shown to have a good oral quality of life, with total OHIP-14 and PIDAQ-23 scores not exceeding 12 and 7.5, respectively, in any of the study groups and subgroups. This indicated that the patients receiving these rehabilitation treatments and having complications did not perceive these negative events as particularly harmful [28].

Furthermore, both treatment outcomes—whether or not complications occurred—appeared to be predictable in terms of enhancing the quality of life of the patients. These results were superior to those reported by Fernández-Ruiz and colleagues, who scored a final overall OHIP-14 of 18.48 [29]. This was probably because patients in the present study—who were interviewed one year after surgery and immediate loading—expressed their satisfaction with the prompt resolution of their primarily cosmetic concerns. In terms of their aesthetic and chewing abilities, patients reported statistically significant improvements. In fact, at the one-year follow-up, 91.7% of patients looked more confident when they smiled; 97.8% and 99.1% of subjects, respectively, did not exhibit relationship problems or feelings of envy towards other people; moreover, 98.8% of participants reported very much better feelings about their appearance. These outcomes were exactly in line with what was found in the literature regarding full-arch prostheses supported by mixed implant approaches (standard, zygomatic, and pterygoid) [29].

Nevertheless, some authors suggested that women were more concerned about their dental health and had higher oral requirements than men [30]. However, other researchers have refuted this assumption: some studies revealed that men prioritized dental appearance more than women [31], while others found that these gender-based differences lacked statistical significance [32,33]. The present study confirmed that the following implantation and immediate loading approach resulted in similar improvements across genders: both males and females experienced equal improvements in dental self-confidence, aesthetic attitude, and social impact.

The analysis of OHIP-14 and PIDAQ-23 scores demonstrated no statistically significant differences between patients with early complications and those without. Consequently, the null hypothesis is accepted, confirming that the occurrence of early complications did not adversely affect patient-reported outcomes related to quality of life and satisfaction. These findings highlight the clinical reliability of the Maxilla-for-All^®^/All-On-X protocols in achieving consistent rehabilitative success, regardless of minor surgical or technical complications.

Maintaining optimal oral hygiene and adhering to a structured maintenance program were critical for the long-term success of implant-supported full-arch rehabilitations, particularly in complex cases involving standard and extra-maxillary implants. While the present study focused on patient-reported outcomes, all participants were enrolled in a professional hygiene recall program, which included regular clinical assessments and reinforcement of individualized oral care instructions. Given the anatomical complexity and increased biomechanical load associated with zygomatic and pterygoid implants, stringent hygiene protocols and routine peri-implant evaluations should be considered an integral part of the rehabilitation protocol to minimize complications and support favorable clinical outcomes as well as in all other full-arch rehabilitation strategies [34].

Although the recruitment of participants for this study was restricted to just three dental clinics, the treatment appears robust and effective across a range of clinical scenarios. A more heterogeneous sample would help generalize these results to other rehabilitation settings. One additional limitation was the variability in implant types and numbers across patients. These variations might have an impact on the present results. However, to better evaluate quality of life and satisfaction of patients following full-arch fixed prostheses supported by conventional, pterygoid, trans-sinus, and zygomatic implants in maxillary sites, long-term clinical studies with larger sample sizes are required.

## 5. Conclusions

Rehabilitation of edentulous atrophic maxillae using full-arch fixed prostheses supported by standard and long implants—including pterygoid, trans-sinus, and zygomatic ones—led to statistically significant improvements in patients’ aesthetic perception and masticatory function. Moreover, these improvements were observed regardless of the occurrence of early surgical or prosthetic complications, as no significant differences in patient-reported quality of life or satisfaction were found between patients with or without such biological and mechanical issues.

## Figures and Tables

**Table 1:** Items of PIDAQ with 0 = not at all, 1 = a little, 2 = somewhat, 3 = strongly, or 4 = very strongly (note that responses for DSC factor were reversed), and OHIP (0 = never, 1 = rarely, 2 = occasionally, 3 = often, 4 = very often), categorized by each factor.

	Times	Preoperative					Postoperative (12 Months)				
factors	PIDAQ-23 items	0	1	2	3	4	0	1	2	3	4
**(reverse)** **Dental** **Self-** **confidence**	1. I am NOT proud of my teeth	0	2	2	12	40	33	21	2	0	0
	2. I DON’T like to show my teeth when I smile	1	3	9	16	27	30	24	2	0	0
	3. I am NOT pleased when I see my teeth in the mirror	2	2	5	17	30	37	17	2	0	0
	4. My teeth are NOT attractive to others	0	1	1	21	33	17	27	9	3	0
	5. I am NOT satisfied with the appearance of my teeth	3	2	4	13	34	25	25	5	0	1
	6. I find my tooth position to be NOT very nice	1	3	7	13	32	32	20	4	0	0
percent (%)		2.1	3.9	8.3	27.4	58.3	51.8	39.9	7.1	0.9	0.3
**Social** **Impact**	7. I hold myself back when I smile so my teeth don’t show so much	13	8	18	11	6	40	14	1	1	0
	8. If I don’t know people well I am sometimes concerned what they might think about my teeth	12	16	15	10	3	39	15	1	1	0
	9. I’m afraid other people could make offensive remarks about my teeth	27	14	10	2	3	46	9	1	0	0
	10. I am somewhat inhibited in social contacts because of my teeth	21	13	17	3	2	47	8	1	0	0
	11. I sometimes catch myself holding my hand in front of my mouth to hide my teeth	20	16	10	7	3	39	16	0	1	0
	12. Sometimes I think people are staring at my teeth	28	14	7	3	4	38	17	1	0	0
	13. Remarks about my teeth irritate me even when they are meant jokingly	35	8	6	5	2	45	10	1	0	0
	14. I sometimes worry about what members of the opposite sex think about my teeth	28	9	12	6	1	50	5	1	0	0
percent (%)		41.1	21.9	21.2	10.5	5.4	76.8	21.0	1.5	0.7	0
**Psychological** **Impact**	15. I envy the nice teeth of other people	5	12	18	13	8	30	23	3	0	0
	16. I am somewhat distressed when I see other people’s teeth	26	17	6	5	2	49	7	0	0	0
	17. Sometimes I am somewhat unhappy about the appearance of my teeth	12	14	21	4	5	53	3	0	0	0
	18. I think most people I know have nicer teeth than I do	9	10	21	10	6	50	6	0	0	0
	19. I feel bad when I think about what my teeth look like	19	10	18	2	7	53	3	0	0	0
	20. I wish my teeth looked better	7	9	5	16	19	51	5	0	0	0
percent (%)		23.2	21.4	26.5	14.9	14.0	85.1	14.0	0.9	0	0
**Aesthetic** **Concerns**	21. I don’t like to see my teeth in the mirror	10	22	12	4	8	54	2	0	0	0
	22. I don’t like to see my teeth in photographs	11	21	14	3	7	52	3	0	1	0
	23. I don’t like to see my teeth when I look at a video of myself	13	18	14	4	7	51	4	0	1	0
percent (%)		20.2	36.3	23.8	6.6	13.1	93.4	5.4	0	1.2	0
factors	OHIP-14 items	0	1	2	3	4	0	1	2	3	4
**Functional** **limitation**	1. Have you had trouble pronouncing any words because of problems with your teeth, mouth or dentures?	7	12	23	12	2	7	24	21	4	0
	2. Have you felt that your sense of taste has worsened because of problems with your teeth, mouth or dentures?	2	14	35	5	0	15	32	6	2	1
**Physical** **pain**	3. Have you had painful aching in your mouth?	0	7	13	35	1	9	13	27	7	0
	4. Have you found it uncomfortable to eat any foods because of problems with your teeth, mouth or dentures?	0	6	20	30	0	6	21	25	4	0
**Psychological discomfort**	5. Have you been self-conscious because of your teeth, mouth or dentures?	4	3	15	29	5	23	25	7	1	0
	6. Have you felt tense because of problems with your teeth, mouth or dentures?	6	4	37	8	1	23	27	6	0	0
**Physical** **disability**	7. Has your diet been unsatisfactory because of problems with your teeth, mouth or dentures?	1	14	16	25	0	8	20	27	1	0
	8. Have you had to interrupt meals because of problems with your teeth mouth or dentures?	4	18	28	5	1	20	27	8	1	0
**Psychological** **disability**	9. Have you found it difficult to relax because of problems with your teeth, mouth or dentures?	5	12	33	6	0	21	31	4	0	0
	10. Have you been a bit embarrassed because of problems with your teeth, mouth or dentures?	4	4	17	24	7	25	26	5	0	0
**Social** **disability**	11. Have you been a bit irritable with other people because of problems with your teeth mouth or dentures?	6	13	27	9	1	29	24	3	0	0
	12. Have you had difficulty doing your usual jobs because of problems with your teeth mouth and dentures?	8	31	10	7	0	42	11	3	0	0
**Handicap**	13. Have you felt that life in general was less satisfying because of problems with your teeth mouth and dentures?	7	13	15	20	1	39	13	4	0	0
	14. Have you been totally unable to function because of problems with your teeth, mouth or dentures?	23	15	12	4	2	37	15	4	0	0
percent (%)		9.8	21.2	38.4	27.9	2.7	38.8	39.4	19.1	2.6	0.1

**Table 2:** Patients’ descriptive statistics.

Time	Preoperative		Postoperative (12 Months)		Wilcoxon Test
PIDAQ-23	mean ± std	[min max]	mean ± std	[min max]	signed-rank *p*-value
Dental Self-Confidence	20.2 ± 4.3	[3–24]	3.5 ± 3.0	[0–12]	**<0.0001**
Social Impact	9.4 ± 7.2	[0–27]	2.1 ± 3.0	[0–18]	**<0.0001**
Psychological Impact	10.5 ± 5.7	[0–24]	1.0 ± 1.3	[0–7]	**<0.0001**
Aesthetic Concerns	4.7 ± 3.6	[0–12]	0.3 ± 1.0	[0–6]	**<0.0001**
PIDAQ tot	44.7 ± 16.6	[11–84]	6.8 ± 5.3	[0–25]	**<0.0001**
OHIP-14	mean ± std	[min max]	mean ± std	[min max]	signed-rank test
Functional limitation	3.6 ± 1.4	[1–7]	2.4 ± 1.3	[0–6]	**<0.0001**
Physical pain	5.0 ± 1.3	[2–7]	3.1 ± 1.5	[0–5]	**<0.0001**
Psychological discomfort	4.4 ± 1.7	[0–8]	1.5 ± 1.3	[0–5]	**<0.0001**
Physical disability	3.8 ± 1.5	[0–7]	2.2 ± 1.3	[0–5]	**<0.0001**
Psychological disability	4.2 ± 1.6	[0–7]	1.3 ± 1.2	[0–4]	**<0.0001**
Social disability	3.0 ± 1.6	[0–6]	0.8 ± 1.1	[0–4]	**<0.0001**
Handicap	3.0 ± 1.7	[0–7]	0.8 ± 1.2	[0–4]	**<0.0001**
OHIP Tot	27.0 ± 7.6	[9–38]	12.0 ± 6.4	[0–28]	**<0.0001**

**Table 3:** Patients’ descriptive statistics in the two groups: with versus without biological plus mechanical complications. Wilcoxon signed-rank test ° and Wilcoxon rank-sum test #. Significance in bold.

Time	Groups	Preoperative			Postoperative (12 Months)			
PIDAQ-23	complications	mean ± std	[min max]	variance	mean ± std	[min max]	variance	*p*-value
Dental Self-confidence	YES	20.6 ± 2.7	[14–24]	7.19	4.1 ± 3.3	[0–12]	10.87	**0.0002 °**
	NO	19.9 ± 5.0	[3–24]	24.59	3.2 ± 2.9	[0–12]	8.27	**<0.0001 °**
*p*-value	YES vs. NO	0.9089 ^#^			0.5554 ^#^			
Social Impact	YES	9.8 ± 6.5	[0–25]	41.67	2.7 ± 4.1	[0–18]	16.91	**0.0013 °**
	NO	9.2 ± 7.6	[0–27]	58.02	1.8 ± 2.3	[0–8]	5.19	**<0.0001 °**
*p*-value	YES vs. NO	0.4988 ^#^			0.9628 ^#^			
Psychological Impact	YES	11.8 ± 5.3	[1–24]	28.14	0.7 ± 0.6	[0–2]	0.70	**0.0002 °**
	NO	9.9 ± 5.8	[0–22]	33.73	1.1 ± 1.5	[0–7]	2.23	**<0.0001 °**
*p*-value	YES vs. NO	0.9281 ^#^			0.9801 ^#^			
Aesthetic Concerns	YES	4.7 ± 3.7	[0–12]	13.35	0.1 ± 0.2	[0–1]	0.05	**0.0004 °**
	NO	4.6 ± 3.7	[0–12]	13.42	0.4 ± 1.2	[0–6]	1.37	**<0.0001 °**
*p*-value	YES vs. NO	1 ^#^			0.7588 ^#^			
PIDAQ tot	YES	44.1 ± 14.6	[26–84]	212.17	7.5 ± 5.7	[1–25]	31.91	**0.0002 °**
	NO	43.6 ± 17.6	[11–84]	308.40	6.5 ± 5.1	[0–19]	26.41	**<0.0001 °**
*p*-value	YES vs. NO	0.7013 ^#^			0.5270 ^#^			
OHIP-14	complications	mean ± std	[min max]	Variance	mean ± std	[min max]	variance	signed-rank test
Functional limitation	YES	4.0 ± 1.3	[2–7]	1.64	2.7 ± 1.4	[1–6]	1.88	**0.0054 °**
	NO	3.4 ± 1.4	[1–6]	1.86	2.2 ± 1.2	[0–4]	1.52	**0.0004 °**
*p*-value	YES vs. NO	0.8521 ^#^			0.8894 ^#^			
Physical pain	YES	5.2 ± 1.2	[2–6]	1.35	3.1 ± 1.6	[0–5]	2.64	**0.0023 °**
	NO	4.8 ± 1.4	[2–7]	1.97	3.1 ± 1.4	[0–5]	2.05	**<0.0001 °**
*p*-value	YES vs. NO	0.2258 ^#^			0.8011 ^#^			
Psychological discomfort	YES	4.2 ± 1.9	[0–7]	3.47	1.3 ± 1.3	[0–5]	1.76	**0.0006 °**
	NO	4.5 ± 1.6	[0–8]	2.68	1.5 ± 1.3	[0–4]	1.55	**<0.0001 °**
*p*-value	YES vs. NO	0.5221 ^#^			0.8147 ^#^			
Physical disability	YES	3.9 ± 1.8	[0–7]	3.11	2.0 ± 1.4	[0–4]	2.00	**0.0080 °**
	NO	3.8 ± 1.4	[1–6]	1.86	2.3 ± 1.2	[0–5]	1.40	**0.0004 °**
*p*-value	YES vs. NO	0.5785 ^#^			0.4190 ^#^			
Psychological disability	YES	4.4 ± 1.7	[0–6]	2.95	1.2 ± 0.9	[0–4]	0.85	**0.0005 °**
	NO	4.1 ± 1.6	[0–7]	2.39	1.4 ± 1.2	[0–4]	1.54	**<0.0001 °**
*p*-value	YES vs. NO	0.5582 ^#^			1 ^#^			
Social disability	YES	3.3 ± 1.8	[0–6]	3.27	1.0 ± 1.2	[0–4]	1.52	**0.0042 °**
	NO	2.9 ± 1.4	[0–6]	2.07	0.8 ± 1.0	[0–4]	0.94	**<0.0001 °**
*p*-value	YES vs. NO	0.9813 ^#^			0.8271 ^#^			
Handicap	YES	3.2 ± 1.8	[0–7]	3.12	0.9 ± 1.2	[0–4]	1.46	**0.0032 °**
	NO	2.8 ± 1.7	[0–7]	2.78	0.7 ± 1.1	[0–4]	1.29	**<0.0001 °**
*p*-value	YES vs. NO	0.8515 ^#^			0.7641 ^#^			
OHIP Tot	YES	28.2 ± 6.9	[9–36]	47.74	12.2 ± 6.1	[2–22]	37.08	**0.0005 °**
	NO	26.3 ± 7.9	[9–38]	63.08	12.0 ± 6.6	[0–28]	43.78	**<0.0001 °**
*p*-value	YES vs. NO	0.7328 ^#^			0.8920 ^#^			

## Data Availability

The original contributions presented in this study are included in the article. Further inquiries can be directed to the corresponding author.

## Abbreviations

The following abbreviations are used in this manuscript:

OHIP	Oral Health Impact Profile
PIDAQ	Psychosocial Impact of Dental Aesthetics Questionnaire
NSAID	Nonsteroidal anti-inflammatory drug
DSC	Dental self-confidence
SI	Social impact
PI	Psychological impact
AC	Aesthetic concern
ANOVA	ANalysis Of VAriance
